# MIS-C: A COVID-19-as sociated condition between hypoimmunity and hyperimmunity

**DOI:** 10.3389/fimmu.2022.985433

**Published:** 2022-10-03

**Authors:** Monica Gelzo, Alice Castaldo, Antonietta Giannattasio, Giulia Scalia, Maddalena Raia, Maria Valeria Esposito, Marco Maglione, Stefania Muzzica, Carolina D’Anna, Michela Grieco, Vincenzo Tipo, Antonio La Cava, Giuseppe Castaldo

**Affiliations:** ^1^ CEINGE-Biotecnologie Avanzate, Scarl, Naples, Italy; ^2^ Dipartimento di Medicina Molecolare e Biotecnologie Mediche, Università di Napoli Federico II, Naples, Italy; ^3^ Dipartimento di Scienze Mediche Traslazionali, Sezione di Pediatria, Università di Napoli Federico II, Naples, Italy; ^4^ Pediatric Emergency and Short Stay Unit, Santobono-Pausilipon Children’s Hospital, Naples, Italy; ^5^ Department of Medicine, University of California, Los Angeles, Los Angeles, CA, United States

**Keywords:** MIS-C, autoimmune diseases, autoinflammatory diseases, cytokines, flow cytometry

## Abstract

Multisystem inflammatory syndrome in children (MIS-C) is a rare, severe complication of COVID-19. A better knowledge of immunological, cellular, and genetic characteristics of MIS-C could help better understand the pathogenesis of the disease and contribute to identifying specific diagnostic biomarkers and develop targeted therapies. We studied 37 MIS-C children at hospital admission and 24 healthy controls analyzing serum cytokines (IFN-α, IFN-β, IFN-γ, IL-6, IL-10, IL-17A, IL-12p70 and TNF), lymphocyte populations by flow cytometry and 386 genes related to autoimmune diseases, autoinflammation and primary immunodeficiencies by NGS. MIS-C patients showed a significant increase of serum IFNγ (despite a significant reduction of activated Th1) and ILs, even if with a great heterogeneity among patients, revealing different pathways involved in MIS-C pathogenesis and suggesting that serum cytokines at admission may help to select the inflammatory pathways to target in each patient. Flow cytometry demonstrated a relevant reduction of T populations while the percentage of B cell was increased in agreement with an autoimmune pathogenesis of MIS-C. Genetic analysis identified variants in 34 genes and 83.3% of patients had at least one gene variant. Among these, 9 were mutated in more patients. Most genes are related to autoimmune diseases like *ATM*, *NCF1*, *MCM4*, *FCN3*, and *DOCK8* or to autoinflammatory diseases associated to the release of IFNγ like *PRF1*, *NOD2*, and *MEF*. Thus, an incomplete clearance of the Sars-CoV2 during the acute phase may induce tissue damage and self-antigen exposure and genetic variants can predispose to hyper-reactive immune dysregulation events of MIS-C-syndrome. Type II IFN activation and cytokine responses (mainly IL-6 and IL-10) may cause a cytokine storm in some patients with a more severe acute phase of the disease, lymphopenia and multisystemic organ involvement. The timely identification of such patients with an immunocytometric panel might be critical for targeted therapeutic management.

## Introduction

Multisystem inflammatory syndrome in children (MIS-C) is a rare, severe complication of COVID-19 (1, 2). It is characterized by non-specific symptoms such as fever, gastrointestinal (vomiting, abdominal pain, diarrhea), conjunctival and mucocutaneous involvement, neurologic alterations (headache, irritability, encephalopathy), and can lead to severe complications including left ventricular dysfunction, cardiovascular shock, and multi-organ failure (3–7). Typically, MIS-C appears in children and adolescents about 2-6 weeks after SARS-CoV-2 acute infection and may represent a delayed, hyperimmune response to SARS-CoV-2 (8). In younger children, MIS-C shares some clinical and laboratory features with pediatric inflammatory multisystemic syndromes like Kawasaki Disease (KD), toxic shock syndrome (TSS), macrophage activation syndrome (MAS), hemophagocytic lymphohistiocitosis (HLH), and other autoinflammatory syndromes (9–11). Increased levels of serum biomarkers like interferon (IFN)-γ, C-reactive protein (CRP), fibrinogen, procalcitonin, D-dimer and ferritin represent disease hallmarks in those patients, but an autoimmune signature was also reported in MIS-C patients (12). In other cases, particularly in adolescents, MIS-C has features of a cytokine storm syndrome, with increased serum cytokine levels, neutrophilia (due to activation of the myeloid compartment) and marked lymphopenia with a pronounced T-cell involvement including T-cell activation (12) - a profile also observed in adult patients with severe acute SARS-CoV-2 infection (13, 14). Also, the finding that only a small subset of previously healthy children without comorbidities develop MIS-C after acute COVID-19 infection seems to suggest a possible role of genetic predisposition in the risk to develop MIS-C. On the other hand, it was recently demonstrated that inborn errors of type I IFN immunity may predispose to life-threatening COVID-19 (15).

Disease features common with other syndromes and the absence of specific therapies for MIS-C have led to borrowing treatments from other disorders, i.e., the use of steroids, intravenous immunoglobulin (Ig) and immunomodulatory agents (16). A better knowledge of the immunological and cellular characteristics of MIS-C patients could help better understand the pathogenesis of this disease, and possibly contribute to identifying specific diagnostic biomarkers and/or develop targeted therapies, to ultimately improve disease management and prognosis. Toward this aim, we performed analyses of large panels of serum proinflammatory biomarkers, flow cytometry, and analyses of gene variants associated with immune responses in MIS-C patients.

## Methods

### Patients

We enrolled 37 children diagnosed at hospital admission with MIS-C according to the definition of the Centre for Disease Control and Prevention (12, 17) between March 2021 and March 2022. Although no data on the SARS-CoV-2 variant are available because the RT-PCR analyses of the nasal swab were negative at admission, during the period of enrollment the Delta variant, i.e., B.1.617.2, was the most frequent for COVID-19 cases in Italy. The study was approved by the Ethical Committee of the University Federico II of Naples. All procedures conformed to the Declaration of Helsinki. Informed consent was obtained from the parent/guardian. The only exclusion criterion was the impossibility to obtain consent (n = 0). MIS-C patients had a median age of 7 years (range: 1–14 years) and 13/37 (35%) patients were females. The healthy control (HC) group included 24 children with a median age of 9 years (range: 5 months - 16 years); 10/24 (42%) were females.

### Lymphocyte populations and serum cytokines analyses

Blood samples were collected at admission in tubes containing EDTA and analyzed for leukocyte count by haemocytometer and then by multi-colour flow cytometry using a FACS Canto II (Becton Dickinson). **Supplemental Table 1** reports the lymphocyte populations studied, the corresponding surface markers, and fluorochromes used for the analyses. For serum cytokine measurements, blood samples were collected in tubes without anticoagulant and levels of IFN-α, IFN-β, IFN-γ, interleukin (IL)-6, IL-10, IL-17A, IL-12p70 and tumor necrosis factor (TNF) were analyzed by automated microfluidic immunoassay cartridges on ProteinSimple Ella (Bio-Techne), in accordance with the manufacturer’s instructions.

### DNA extraction for next-generation sequencing

Genomic DNA (gDNA) was isolated from peripheral blood samples (in EDTA) using the robotic workstation MagPurix Systems (Resnova) for the purification of nucleic acids, according to the manufacturer’s instructions. The quality of DNA samples was assessed by TapeStation system (Agilent Technologies) and only gDNA samples with a DNA integrity number (DIN) >6 were used for NGS analysis. DNA quantity was evaluated using the Qubit dsDNA BR and HS assays kits (Life Technologies).

### NGS custom panel design, NGS library preparation and sequencing

NGS analyses were performed using a panel of 386 genes related to autoimmune diseases, autoinflammation and primary immunodeficiencies (**Supplemental Table 2**). For each gene, we analyzed the entire coding regions, 50 bp in each of the intronic boundaries and promoter, for a total target size of about 1.5 Mb. By using a customized clinical exome, we targeted >6,000 genes on which we filtered only genes of interest in bioinformatic analyses. Briefly, a total of 100 ng of gDNA was processed with the SureSelect XT-HT Target Enrichment system using the Magnis NGS Prep System (Agilent Technologies) following the manufacturer’s protocol. Next, SureSelect-enriched dual-indexed NGS samples were pooled together for multiplexed sequencing. Sequencing reactions were carried out on the NextSeq 500 instrument (Illumina, USA) using a PE 150 × 2 High-Output flow cell, running 16 samples for each sequencing run to obtain an average coverage of about 200× (>95% of the gene’s target nucleotides are covered at >100 reads, with mapping quality score (MQ > 30) reads); 99% of the analyzable target regions were covered by at least 50X.

### NGS data analysis

To perform alignments, variant calling, and quality filtering, we used the Alissa Align & Call v5.2.10 tool (Agilent Technologies), using the genome build hg38 as a reference. Variant filtering and interpretation were done using Alissa Interpret v5.2.6 CE IVD software (Agilent Technologies). Bioinformatics predictions of the variant’s effects were performed using SIFT [http://sift.jcvi.org] and PolyPhen-2 (http://genetics.bwh.harvard.edu/pph2). Further predictions were assessed using the Mutation Taster tool and VarSome [https://varsome.com/variant/hg38] and the CADD score, excluding variants with a score <20 (18). Variant’s classification was performed following the American College of Medical Genetics and Genomics (ACMG) guidelines.

### Statistical analysis

The Shapiro–Wilk test was applied to evaluate the normality of distributions. Comparisons between two groups were evaluated by Mann-Whitney U test. Correlations between variables were evaluated using Spearman correlation analysis. For the statistical analysis of values below the limits of sensitivity, concentrations were estimated using the formula of limit of sensitivity/√2 (19). Statistical analyses were performed by SPSS (version 27, IBM SPSS Statistics). Graphics were done using KaleidaGraph software (version 4.5.4, Synergy, Reading, PA, USA). P values <0.05 were considered significant.

## Results

### Serum biomarkers

**Figure 1:**
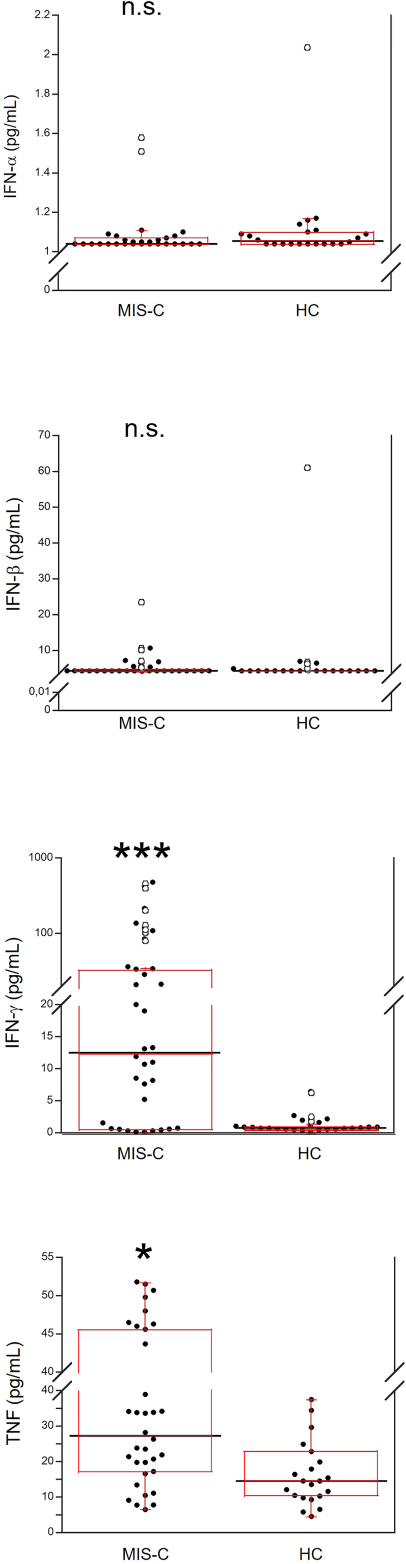
Comparison of serum values of IFN-α, IFN-β, IFN-γ and TNF in 37 patients with MIS-C and in 24 HC. The black line represents the median value. The red box represents the interquartile range. The blank circles correspond to the outliers and the black circles side by side to these correspond to duplicates *p < 0.01; ***p < 0.0001; n.s.: not significant (p > 0.05).

Demographics and frequency of clinical features of the 37 patients with MIS-C are reported in **Table 1**. **Figure 1** shows the serum levels of IFN-α, IFN-β, IFN-γ and TNF in the 37 patients with MIS-C and 24 HC. Levels of IFN-α and IFN-β were not significantly different between the two groups, while serum IFN-γ was significantly higher in MIS-C patients than HC (p<0.0001); 6 of 37 (16.2%) MIS-C patients had IFN-γ levels >100 pg/mL. Serum TNF was also significantly higher in MIS-C patients than HC (p<0.01); 3 of 37 (8.1%) MIS-C patients had TNF levels >50 pg/mL (**Table 2**).

**Table 1:** Demographics and clinical features of the 37 patients with MIS-C.

Age in years, median (range)	7 (1-14)
Males, n (%)	24 (65)
Fever, n (%)	37 (100)
Organ system involvement, n (%)	
-gastrointestinal	32 (86)
-cardiac	26 (70)
-skin	24 (65)
-coagulation	23 (62)
-circulation	5 (13)
-neurological	5 (13)
-musculoskeletal	5 (13)
-respiratory	1 (3)
-renal	1 (3)

**Table 2:** MIS-C patients with the highest levels of serum cytokines.

Cytokines serum levels (pg/mL)	IFNγ >100	TNF >50	IL-6 >200	IL-17A >30	IL-10 >100
Patient #					
1			461		105
2	109	51.5	288	39.4	509
3	137		271		
4	119				150
5				74	
6	215		730		311
7		51.8			
8		50.7			
9			266		
10			271		122
11	477		476		174
12	420		409		
13					140
14					163

**Figure 2:**
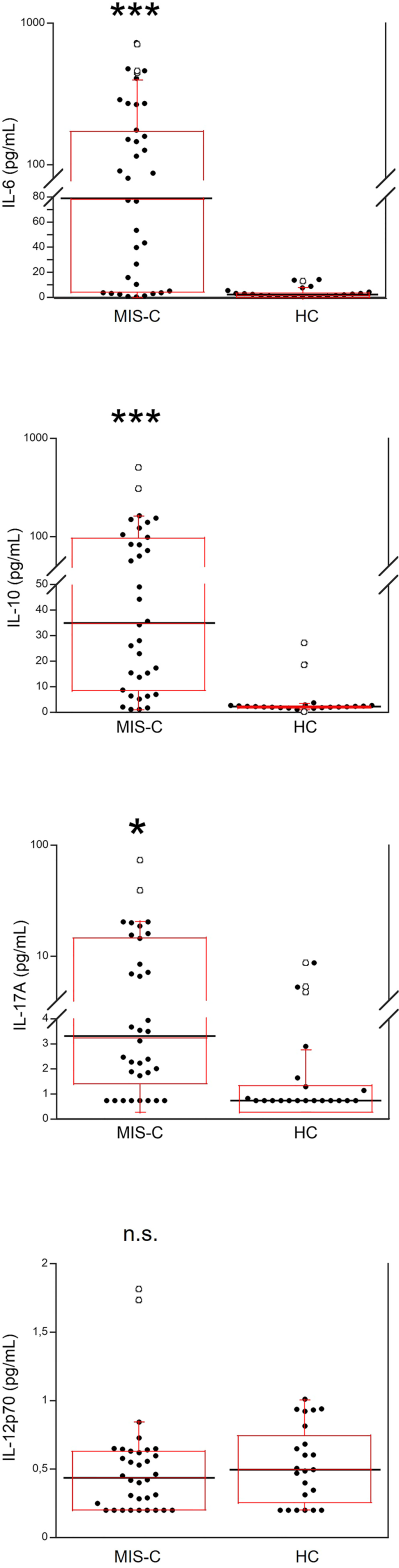
Comparison of serum levels of IL-6, IL-10, IL-17A and IL-12p70 in 37 patients with MIS-C and in 24 HC. The black line represents the median value. The red box represents the interquartile range. The blank circles correspond to the outliers and the black circles side by side to these correspond to duplicates *p < 0.01; ***p < 0.0001; n.s.: not significant (p > 0.05).

Serum IL-6, IL-10, IL-17A and IL-12p70 were also measured (**Figure 2**). IL-6 was significantly higher in MIS-C patients, as compared to HC (p<0.0001); 8 patients’ (21.6%) had IL-6 levels >200 pg/mL (**Table 2**). Serum IL-10 was significantly higher in MIS-C patients (p<0.0001) than in HC; 8 patients (21.6%) had IL-10 levels >100 pg/mL. IL-17A (p<0.01) was also higher in the MIS-C patients; 2 patients (5.4%) had IL-17A levels >30 pg/mL (**Figure 2** and **Table 2**). No significant differences were observed between MIS-C patients and HC for IL-12p70 (**Figure 2**).

### Immunophenotyping

**Figure 3:**
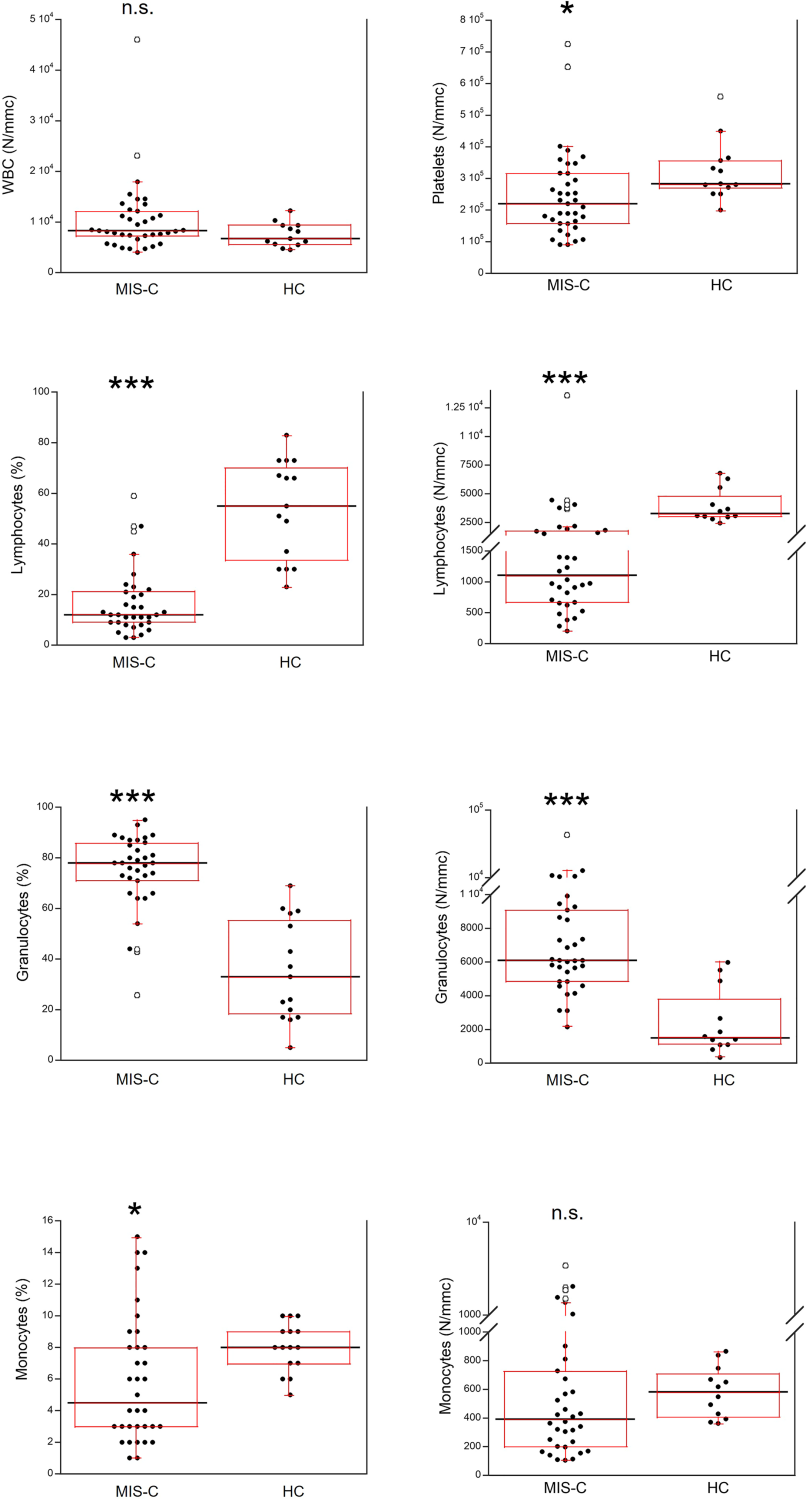
Comparison of the complete blood count (CBC) in 37 patients with MIS-C and in 24 HC. The black line represents the median value. The red box represents the interquartile range. The blank circles correspond to the outliers and the black circles side by side to these correspond to duplicates *p < 0.01; ***p < 0.0001; n.s.: not significant (p > 0.05).

The total number of white blood cells (WBC) in the MIS-C patients did not differ from that found in HC (**Figure 3**). However, platelet counts were lower (p<0.01) in MIS-C patients, which also had relative and absolute lymphopenia (p<0.0001 for both) and neutrophilia (p<0.0001 for both). A relative reduction of monocytes in MIS-C patients (p<0.01) was not accompanied by differences in absolute monocyte numbers.

**Figure 4:**
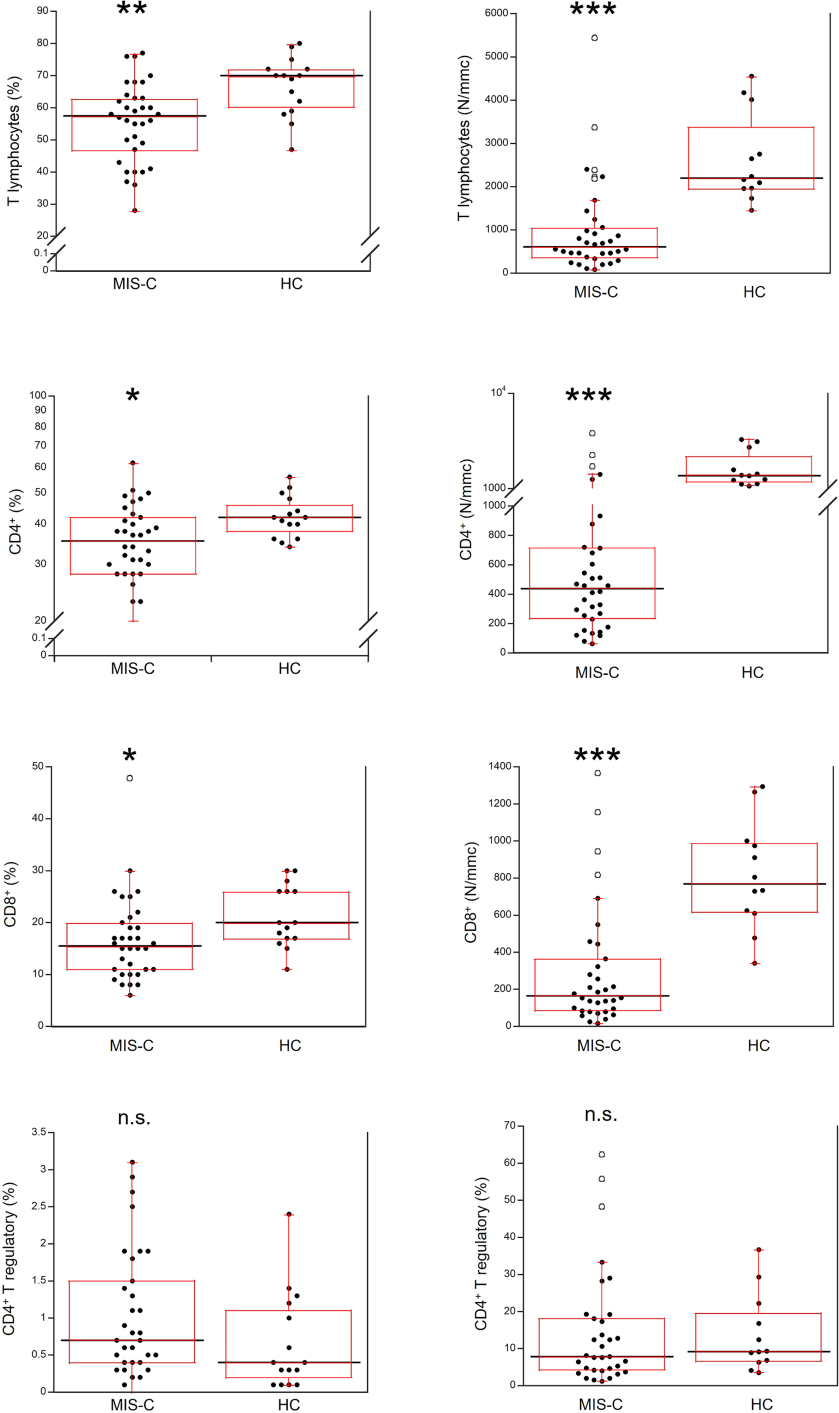
Comparison of total T lymphocytes and T lymphocyte populations in 37 patients with MIS-C and in 24 HC. The black line represents the median value. The red box represents the interquartile range. The blank circles correspond to the outliers and the black circles side by side to these correspond to duplicates *p < 0.01; **p < 0.001; ***p < 0.0001; n.s.: not significant (p > 0.05).

Relative and absolute numbers of T cells were significantly reduced in MIS-C patients (p<0.001 and p<0.0001, respectively), including CD4+ and CD8+ T cells (in both cases p<0.01 for percentages, and p<0.0001 for absolute numbers). CD4+ T regulatory cells (Tregs) were not significantly different between MIS-C patients and HC both as percentage and absolute numbers (**Figure 4**).

**Figure 5:**
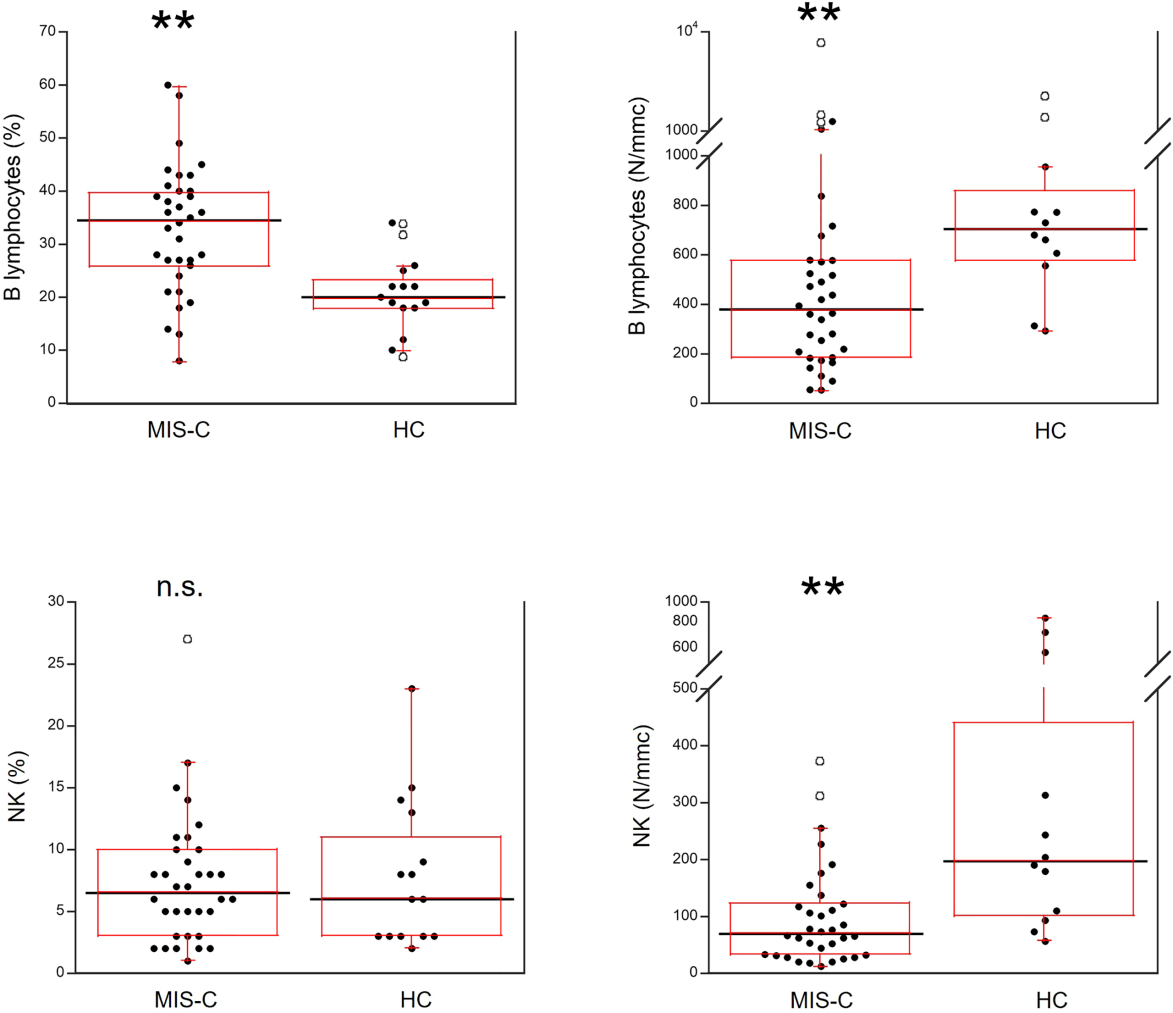
Comparison of B and NK lymphocytes in 37 patients with MIS-C and in 24 HC. The black line represents the median value. The red box represents the interquartile range. The blank circles correspond to the outliers and the black circles side by side to these correspond to duplicates **p < 0.001; n.s.: not significant (p > 0.05).

As shown in **Figure 5**, for B cells, relative numbers were increased in MIS-C patients (p<0.001) but absolute numbers were reduced (p<0.001). The percentages of NK cells did not differ between MIS-C patients and HC, while the absolute numbers were lower in the former (p<0.001).

**Figure 6:**
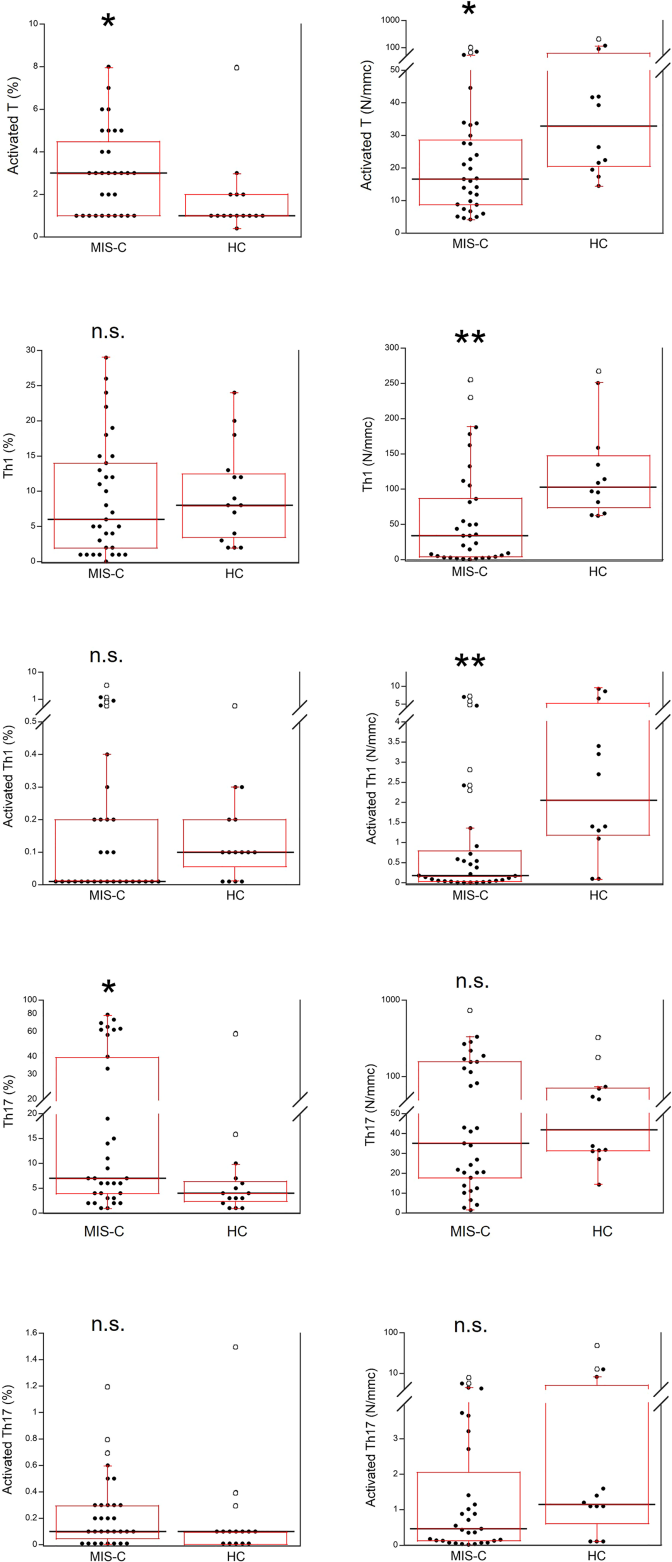
Comparison of T activated, activated Th1 and Th17 lymphocytes in 37 patients with MIS-C and in 24 HC. The black line represents the median value. The red box represents the interquartile range. The blank circles correspond to the outliers and the black circles side by side to these correspond to duplicates *p < 0.01; **p < 0.001; n.s.: not significant (p > 0.05).

Activated T cells were significantly increased as percentages (p<0.01) but reduced as absolute numbers (p<0.01) in the MIS-C patients as compared to HC. For the T cell subsets, percentage numbers of Th1 cells and activated Th1 cells were not significantly different between MIS-C patients and HC but both types were reduced in the former group as absolute numbers (p<0.001 for both). On the contrary, percentages of Th17 cells were increased in MIS-C patients (p<0.01), while absolute numbers of Th17 did not differ from HC. Activated Th17 cells did not differ between MIS-C patients and HC (both as percentages and absolute numbers) (**Figure 6**).

### Genetic analyses

NGS was used to analyze gene variants of 386 genes encoding proteins related to immunodeficiencies, autoimmune and inflammatory diseases (**Supplementary Table 2**) in 30 MIS-C patients. **Supplemental Table 3** summarizes the results reporting the gene and corresponding MIM number; the variant (named according to the HGVS nomenclature), and RS number of the variant. For each variant, the frequency is reported according to the GnomAD_exome and ExAC repositories and to the frequencies previously assessed in 380 patients referred to our laboratory for immunodeficiency, autoimmune or autoinflammatory diseases. Prediction in silico of the pathogenicity of each variant was obtained using three tools - PolyPhen, SIFT, Mutation tester. All variants in which at least two of the three tools excluded pathogenicity were excluded. Also, each variant was assessed using the CADD tool (18) and variants with a score <20 were excluded.

The analysis identified variants in 34 genes; 25/30 (83.3%) of the MIS-C patients had at least one variant in one of the genes, and 5/30 (16.7%) of the patients did not display any gene variant. Some genes were mutated in more patients (i.e., *PRF1*: 5 cases; *ATM*: 3 cases; *DOCK8*: 3 cases; *NCF1*: 2 cases; *SLC37A4*: 2 cases; *MCM4*: 2 cases; *NOD2*: 2 cases, among which 1 double heterozygous for two variants; *FCN3*: 2 cases). All gene mutations found in more than 1 patient were different except for the 5 patients with the *PRF1* mutations (all had the Ala91Val mutation), and the 2 patients with *FCN3* mutations (both had the Leu117fs mutation). Fourteen variants had not been reported previously; all the other variants are rare.

## Discussion

We report that patients with MIS-C at hospital admission had a significant increase of serum pro-inflammatory biomarkers including type II IFN (IFN-γ) but not type I IFNs (IFN-α and IFN-β), differently from acute SARS-CoV-2 infection where type I IFN responses appear as possibly more relevant, confirming the distinct immunopathological signature between acute COVID-19 and MIS-C (20). While it has been reported that in patients with MIS-C the Th1 pathway [i.e., IFN-γ-related (21)] is enhanced and the Th2 responses are suppressed (19), we found a reduced number of activated Th1 cells which, in most MIS-C patients were almost absent (although such reduction was transient, see below). In agreement with previous observations (12, 22, 23), we also found in MIS-C patients increased IL-10, IL-6 and IL-17, the latter two related to the Th17 cell pathway and increased in hyperinflammatory, febrile status (21). Those alterations were variable among the MIS-C patients, i.e., only one third at hospital admission displayed serum cytokine levels typical of the cytokine storm (12) observed in adults with severe acute COVID-19 (13, 14). Moreover, not all cytokines showed the same trends, indicating the activation of different inflammatory pathways in different MIS-C patients. As such, these results suggest consideration of the serum cytokine profiles in patients with MIS-C at hospital admission for possible early selection of the most relevant cytokine pathway(s) to possibly target therapeutically.

Another consideration relates to the finding that MIS-C patients have thrombocytopenia, granulocytosis and lymphopenia (23). Thrombocytopenia is not due to direct viral damage to platelets (24) or to a suppression of bone marrow production since other blood cell lineages are normally produced. More likely, it depends on autoimmune destruction or on enhanced platelet activation and consumption (20) and this, together with the evidence of neutrophil activation (25), suggests considerations of anticoagulant therapies in selected MIS-C patients. In this context, increased neutrophil production and activation is a central component of the immune response against SARS-CoV-2 infection and contributes to severe COVID-19 in adults (26). Such activation was also found in children with COVID-19 and MIS-C (12, 20) and possibly is driven by cytokines (12).

We also found a reduction of the absolute numbers of total lymphocytes and T, B and NK cells, in agreement with previous studies (12, 23) that suggested a possibility of cell extravasation and consumption - also triggered by cytokines (27), like in severe acute COVID-19 (13). For CD4+ and CD8+ T cells we also found significantly reduced percentages. Whether this might be due to enhanced cell death by apoptosis as we (13) and others (28) observed in adult severe COVID-19 needs to be investigated. We also found a reduction of activated Th1 cells but not of activated Th17 cells, which was transient since about half of the MIS-C patients analyzed after two weeks from admission had a restoration of the numbers of activated Th1 (data not shown). The reduction of activated Th1 might have been induced by IL-6, that inhibits Th1 polarization. While the increased percentage of B cells suggested mechanisms of relative maintenance, in spite of their reduction in absolute numbers. This needs to be investigated further because B cell responses and the production of autoantibodies may contribute to MIS-C (12), even if the rapid resolution of MIS-C suggests an involvement of short-lived cells (23).

Importantly, in most MIS-C patients we found pathogenic variants of genes involved in immunodeficiencies, autoimmune and autoinflammatory diseases (**Supplemental Table 3**). Although the pathogenesis of such diseases is different, possible similarities might be seen in the observation that most patients with congenital immunodeficiency frequently develop autoimmune diseases (29), and that autoinflammatory and autoimmune events during COVID-19 contribute to multisystemic inflammatory diseases (30, 31). In this context, Zhang Q et al. recently demonstrated that mutations in 8 genes related to type I IFN predispose to life-threatening COVID-19 pneumonia (15). Also, autoimmune biomarkers (12,20) and biochemical and clinical evidence of hyperinflammation represent hallmarks of MIS-C. In our cohort of MIS-C patients, we found potentially pathogenic variants in 34 genes related to immune and inflammatory disorders, and more than 80% of patients had one or more variant. Such variants were predicted as likely pathogenic by different tools. All are rare and some were not previously detected in comparisons with repositories for the general population. As detailed in **Supplemental Table 3**, variants of such genes predispose to autoimmune cytopenia, like *ATM* - which was mutated in 3 MIS-C patients. Other were previously related to SLE, like *NCF1*, that we found mutated in two MIS-C patients and previously in 5 autoimmune patients (unpublished results). Mutations in MIS-C patients were found also in other genes related to autoimmune diseases like *MCM4* and *DOCK8* (both mutated in 3 MIS-C patients) and *FCN3*, mutated in two MIS-C patients and previously found mutated in patients with immunodeficiency and autoimmunity by our group, all bearing the Leu117fs pathogenic mutation (unpublished results).

We hypothesize that elevated pro-inflammatory cytokines might act in concert with those predisposing backgrounds, considering their increase in most MIS-C patients and possible sustenance by gene variants identified in the MIS-C patients and previously in autoinflammatory diseases associated with enhanced production of IFN-γ (32) [such as HLH or Blau syndrome (33)]. Those genes include *PRF1*, that we found mutated in 5 patients with MIS-C and previously in 20 patients with autoinflammatory diseases, all bearing the pathogenic Ala91Val mutation that seems to be peculiar to Southern Italy and *NOD2* (mutated in two MIS-C patients - one of which compound heterozygous for two different mutations). Our results are in agreement with a recent report documenting HLH associated gene (including *DOCK8* and *PRF1*) mutations identified in children with MIS-C (34). Of interest, one MIS-C patient was heterozygous for three mutations in the *MEFV* gene. *MEFV* mutations are responsible for familial Mediterranean fever, that in turn associates with elevated IFN-γ responses (35). These three mutations were defined as pathogenic with a different concordance between the three tools, and their pathogenicity might increase when present together.

While we think that when considered alone, the gene variants in MIS-C patients – even if present in many patients – might not have a determining impact on the development of the disease, the finding that most patients had variants of more genes might lead to suggest possible predisposing effects, e.g through trans-heterozygosity (36–38). In other words, we acknowledge that our low number of cases and the heterogeneity of the involved genes represent a limitation to define whether the mutated gene(s) contributed to an unbalanced immune homeostasis causing aberrant immune responses that would promote hyperinflammation and autoimmunity (39). Yet the mounting evidence that children with sepsis or other critical illness (40) have a higher prevalence of potentially pathogenic variants in genes related to immunity suggests that a predisposing genetic background can facilitate the development of pathologic conditions triggered by infectious agents, with subsequent disease exacerbations and severe complications.

To conclude, we report that MIS-C has characteristics of both hypo- and hyper-reactive immune dysregulation, with genetic variants that can predispose to pro-inflammatory events that may promote MIS-C-induced tissue damage and self-antigen exposure to the immune system. IFN-γ activation and cytokine responses (mainly IL-6 and IL-10) may cause a cytokine storm in some patients with a more severe acute phase of disease and lymphopenia and multisystemic organ involvement. The timely identification of such patients with an immunocytometric panel might be critical for therapeutic intervention and a targeted clinical management of MIS-C.

## Data availability statement

The data presented in the study are deposited in the NCBI SRA repository, accession number PRJNA883706.

## Ethics statement

The studies involving human participants were reviewed and approved by Ethical Committee of the University Federico II of Naples. Written informed consent to participate in this study was provided by the participants’ legal guardian/next of kin.

## Author contributions

Design of the work: GC. Methodology, investigation and data analysis: MGe, GC, AG, GS, MR, ME, MM, SM, CDA, MGr and VT. Manuscript writing and validation: ALC and GC. All authors contributed to the article and approved the submitted version.

## Funding

This work was supported by the Italian Ministry of University and Research (PRIN 2020, code 20209TB4AX) and by Regione Campania (CEINGE-TASK-force COVID-19, code D64I200003800, and POR Campania FESR 2014-2020).

## Acknowledgments

We thank the CEINGE TASK-FORCE-2022 COVID19. Realizzazione di servizi di ricerca per la lotta contro il COVID 19. Regione Campania (DGR n. 504 del 10/11/2021). CUP n. D63C22000570002, and the staff of CEINGE-Biotecnologie Avanzate, that hosts the scientific activity of the project.

## Conflict of interest

The authors declare that the research was conducted in the absence of any commercial or financial relationships that could be construed as a potential conflict of interest.

## Publisher’s note

All claims expressed in this article are solely those of the authors and do not necessarily represent those of their affiliated organizations, or those of the publisher, the editors and the reviewers. Any product that may be evaluated in this article, or claim that may be made by its manufacturer, is not guaranteed or endorsed by the publisher.
